# Deep Venous Aberration

**DOI:** 10.7759/cureus.13782

**Published:** 2021-03-09

**Authors:** Kishore Karri, Tushi Singh, Nishant Tripathi, Kavya Sudanagunta, Pradeep Yarra

**Affiliations:** 1 Internal Medicine, University of Kentucky, Lexington, USA; 2 Internal Medicine, University of Kentucky, New York, USA; 3 Hospital Medicine, University of Kentucky, Lexington, USA; 4 Radiology, University of Kentucky, Lexington, USA

**Keywords:** deep venous thrombosis, may-thurner's syndrome

## Abstract

Deep venous thrombosis is a common medical diagnosis. Estimates suggest 60,000 to 100,000 deaths annually from deep venous thrombosis and related complications in the United States of America. The diagnosis is often straightforward using a combination of clinical features and ultrasonography. Once confirmed, the treatment is straightforward as well - anticoagulation. However, we might come across a case where despite the prescribed treatment, there is little clinical improvement. There could be myriad reasons for this. We wish to share our experience with one such treatment failure and how we were able to narrow down the etiology to an anatomical defect. Eventually, we were able to offer curative treatment with vessel stenting. This case refreshed our medical knowledge and we hope to do the same for our colleagues.

## Introduction

May-Thurner syndrome (MTS) is the development of iliofemoral deep venous thrombosis (DVT) due to extrinsic compression of the left common iliac vein between the right common iliac artery and lumbar spine. Latest studies show that compression (in the form of spurs) greater than 70% increases the likelihood of left-sided deep venous thrombosis [1]. Most people are clinically asymptomatic but the occurrence of a risk factor like pregnancy, surgery, or malignancy can precipitate a DVT [2]. We want to share a clinical experience where the anatomical reason for a left-sided DVT was identified and successfully treated.

## Case presentation

A 54-year-old female presented with worsening left leg pain and swelling for one day. On presentation, her oral temperature was 36.7 degrees Celsius, heart rate of 76/minute, blood pressure of 129/88 mmHg, and respiratory rate of 18/minute with oxygen saturation of 99% on room air. She was in moderate discomfort due to pain and a physical exam was remarkable for erythematous swelling in the left leg extending from the ankle up to the pelvis. The entire left limb was tender to palpation with feeble distal pulses. 

The patient’s medical history was significant for similar complaints about 30 years ago when she was on oral contraceptives. She stopped them on medical advice and remained well. Eight months prior to the current presentation, she was diagnosed with DVT. There were no risk factors identified at the time. Laboratory workup for procoagulant disorders was negative. She was initiated on warfarin while being bridged with low molecular weight heparin (LMWH). Her International Normalized ratio (INR) remained therapeutic through this time. About three months prior to the current presentation she was diagnosed with endometrial cancer and within a month of diagnosis, she underwent a total abdominal hysterectomy. The perioperative anticoagulation was managed with LMWH and warfarin was resumed on discharge with bridging. The current presentation was 90 days after her surgery. 

**Figure 1:**
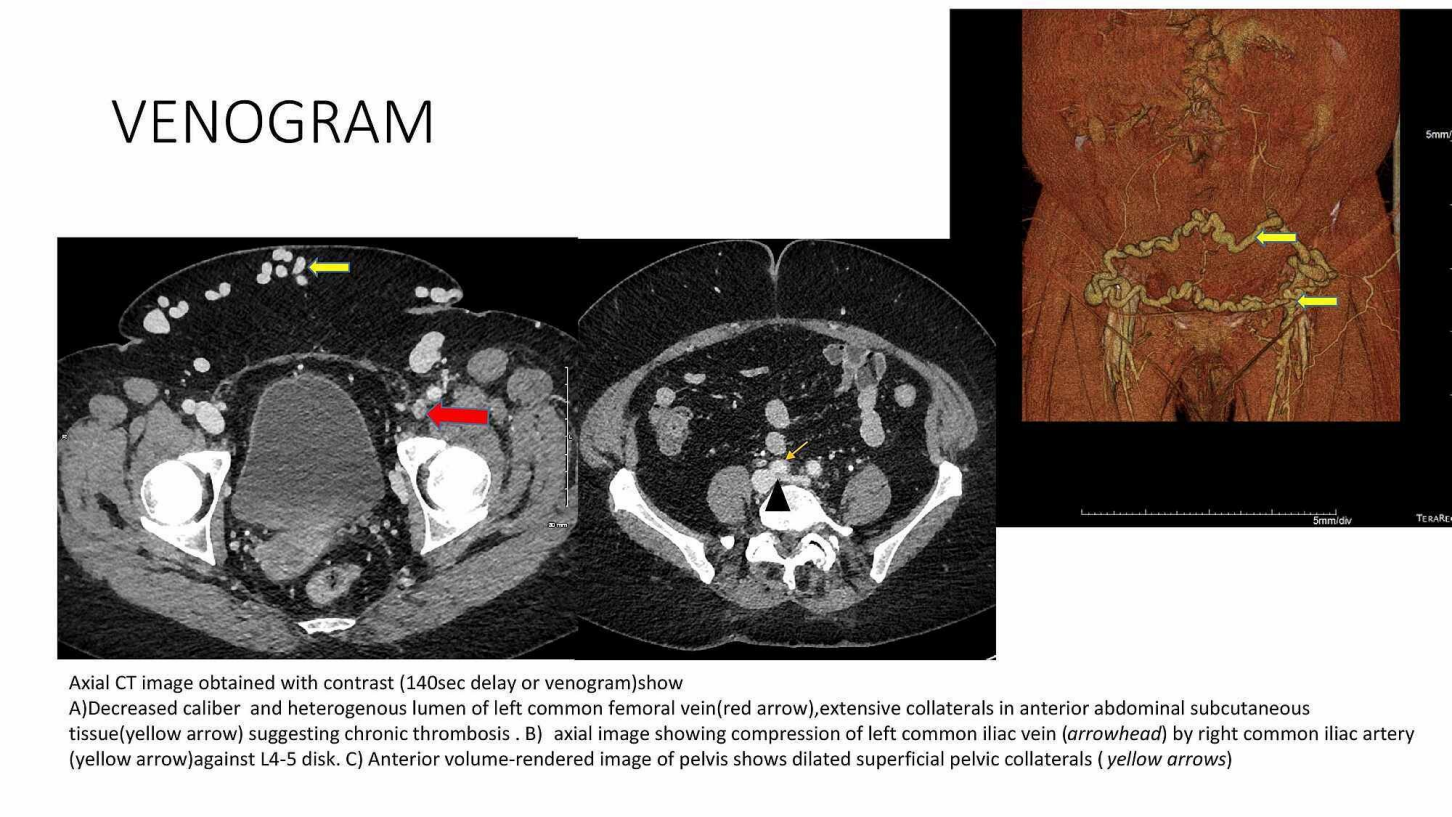
Venogram on presentation

In the current admission, hemoglobin was 9.8 g/dl, white blood cell count was 8670, and platelet count of 188,000 cells per microliter respectively. Her INR was 2.4. All these values were close to her baseline values. Her electrolytes were within normal limits and creatinine was 1.1 mg per deciliter. A venous duplex showed an acute occlusive DVT in the left lower extremity with a superficial thrombosis in the greater saphenous vein. A computed tomography (CT) with contrast of the abdomen and pelvis showed a persistent clot in the left femoral vein which had extended proximally as well as distally (Figure 1). Also noted were numerous venous collaterals. These features were consistent with an acute on chronic DVT. Upon reviewing the details it was clear that she had a recurrence or persistence of the DVT while being appropriately anticoagulated. There was no suggestion of recurrent cancer and she had remained physically active at her farm after the surgery. This prompted a search for possible anatomical reasons for recurrent DVT. The vascular surgery team performed venography in a nearly occluded iliofemoral vein and confirmed our suspicion of MTS. After stenting of the left common iliac vein, there was noticeable improvement in her symptoms within four weeks. At our last contact with the patient, nearly 22 months after the procedure, she remained symptom-free. 

## Discussion

MTS was first described by Virchow in 1841 [3]. May and Thurner noticed the anatomical variant in 22% of the cadaver studies [4]. The true prevalence is still unknown. It ranges from 2-5% as per a few studies [5,6] Autopsy studies suggest a prevalence between 14-32% [5], radiology studies looking at the left lower extremity DVT alone suggest a prevalence from 22% to 76% [5]. A recent case series and extensive discussion by Katalin Mako et al [7] shows how it is often underestimated and needs a high index of suspicion for diagnosis. 

It is classically found in women between the age group of 20 to 50 and studies show it's twice as common in females than males [8,9]. May and Thurner theorized that the pulsations of the right iliac artery overlying the vein led to the development of a “spur” in the wall of the vein. This repeated venous trauma leads to the accumulation of elastin and collagen, contributing to spur formation which leads to partial venous obstruction kicking off the Virchow's triad and resulting in a thrombus [10]. Interestingly, a study found that 67% of the subjects with chronic iliac vein thrombus screened prior to treatment had a hypercoagulable disorder [11]. Left iliac vein compression is the most common variant seen in May-Thurner syndrome; literature review showed other variants as well. Compression of the left common iliac vein by the left internal iliac artery, [12] compression of the right common iliac vein by the right internal iliac artery, [13] compression of the inferior vena cava by the right common iliac artery [14], and right-sided May-Thurner syndrome in a patient with a left-sided inferior vena cava [15] have all been described. 

Most of the studies on May-Thurner syndrome conclude that the prevalence is often underestimated. In the extensive literature review, we find that it is likely from atypical presentation and lack of classical risk factors (young female, use of oral contraceptives, pregnancy) in most cases. It is important to remember that the gold standard for investigation, a venogram or an intravenous ultrasound is not routinely performed to clinch the diagnosis. It is important to diagnose and treat this condition to avoid complications of pulmonary embolism (PE) [7,16] and post-thrombotic syndrome [2, 7]. Our patient had the risk factor of malignancy and this likely diverted attention away from MTS. However, when she presented with symptoms of acute DVT while on anticoagulation, we pursued the diagnosis. 

A Doppler ultrasound is usually the first line of investigation. However, technical difficulty in imaging the iliac vein limits its utility [2]. CT Venography is an excellent tool for evaluation of the structures surrounding the vessel but often overestimates the degree of compression [17]. Magnetic resonance venography is limited by cost and variability in venous compression over time [18]. Venography with intravascular US (IVUS) are the gold standard to diagnose MTS. IVUS provides details of the vessel lumen and vessel wall [19]. It can also assist in guiding management during thrombolysis and stent placement. It eliminates the risk of contrast allergies and nephropathy. Our patient underwent a doppler ultrasound followed by a CT venogram which was very supportive of our diagnosis as illustrated in the case presentation. Following this, the vascular surgery team decided to perform a venogram during which the iliac vein compression was established and a stent was placed in the same sitting. 

Once a DVT is confirmed, anticoagulation must be initiated. There are no specific guidelines for the duration of anticoagulation in MTS. It is suggested that the DVT guidelines be followed [20]. Direct oral anticoagulants are recommended as the first-line. If vitamin K antagonists are chosen, the target INR should be 2-3. Long-term anticoagulation has the benefit of preventing complications such as PE. This should be weighed against the risk of bleeding. The vascular societies recommend endovascular management. Patients with acute thrombosis undergo catheter-directed thrombolysis followed by stent placement to correct the luminal obstruction [9]. Our patient underwent the venogram a few weeks after the diagnosis. She had only a mechanical clot removal followed by stent placement. 

There is an ongoing study by Van Vuren et al (registered with British Medical Journal (BMJ) Open in Jan 2017) to compare outcomes with conventional treatment versus stenting in MTS. These results will likely pave the way for establishing a gold standard for the treatment of MTS. Our patient noted improvement in her swelling and thus her quality of life over four weeks after the procedure. Our last contact was 22 months following the procedure when she was symptom-free. 

## Conclusions

MTS is established to be underrecognized. Based on our clinical experience, we suggest that any patient with recurrent proximal DVT be considered for evaluation of MTS. Early recognition can significantly reduce morbidity. With optimal management, MTS has a good prognosis. The anticoagulation management should be tailored to each patient and every patient should get a thrombophilia screen as a part of this workup. 
